# Neglected zoonotic helminthiases in wild canids: new insights from South America

**DOI:** 10.3389/fvets.2023.1235182

**Published:** 2023-08-11

**Authors:** Manuel Uribe, Jan Brabec, Jenny J. Chaparro-Gutiérrez, Carlos Hermosilla

**Affiliations:** ^1^Biomedical Research Center Seltersberg (BFS), Institute of Parasitology, Justus Liebig University Giessen, Gießen, Germany; ^2^CIBAV Research Group, Veterinary Medicine School, Universidad de Antioquia, Medellín, Colombia; ^3^Institute of Parasitology, Biology Centre of the Czech Academy of Sciences, České Budějovice, Czechia

**Keywords:** neglected, zoonosis, wild canids, Neotropics, dipylidiasis, lagochilascariosis, sparganosis

## Abstract

The global threat of neglected tropical diseases (NTDs) constitutes a public health issue in underdeveloped countries. Zoonotic helminthiases are the most common human NTD agents in developing countries in sub-Saharan Africa, Asia, and the Americas, causing a global burden of disease that exceeds that of more recognized infectious diseases such as malaria and tuberculosis. Wild canids are well-known mammals that act as natural reservoirs of zoonotic-relevant helminthiasis worldwide, thus playing a pivotal role in their epidemiology and transmission to humans. Here we evaluate the occurrence of zoonotic gastrointestinal helminths in two Neotropical wild canid species from the Amazonian and Andean regions of Colombia, i.e., the bush dog (*Speothos venaticus*) and the crab-eating fox (*Cerdocyon thous*). We recovered tapeworm proglottids from bush dog fecal samples and identified them molecularly as the canine-specific lineage of *Dipylidium caninum* by using cytochrome c oxidase subunit I (*cox1*) gene sequences. Moreover, examination of a crab-eating fox during necropsy revealed the presence of non-embryonated eggs of the neglected nematode *Lagochilascaris cf. minor*, in addition to eggs and gravid proglottids of the cestode *Spirometra mansoni*. These findings represent the first report of zoonotic-relevant cestodes, i.e., *D*. *caninum* (“canine genotype”), *S. mansoni*, and the nematode *L. cf. minor*, in bush dogs and crab-eating foxes as final hosts. The occurrence of these zoonotic helminthiases in wild canid species calls for regular monitoring programs to better understand the epidemiology and transmission routes of neglected dipylidiasis, lagochilascariosis, and sparganosis in South America.

## 1. Introduction

Zoonoses account for approximately 60% of emerging human infectious diseases, and among these, up to 70% are wildlife-derived pathogens (1, 2). In addition, the global threat of neglected tropical diseases (NTDs) constitutes a public health issue in underdeveloped countries in sub-Saharan Africa, Asia, and the Americas. Among NTDs, zoonotic helminthiases are the most common human pathogens, causing a global burden of disease exceeding that of better-known infectious diseases such as malaria and tuberculosis. On a global scale, helminth infections account for over 75% of disability-adjusted life years lost. However, many of them have fallen into oblivion as neglected diseases (3, 4). Wild canids are well-known natural reservoirs of zoonotic parasites (5–10), which include numerous helminth species, and thus play a pivotal role in the life cycle, epidemiology, and transmission routes of human infections (11–14). The forested tropical regions with high mammalian species richness are facing the emergence of zoonotic disease hotspots under ongoing land use changes, giving rise to an increased disease transmission risk at the human-animal interface (15, 16). Lower-latitude developing countries (e.g., Neotropical territories) have a concentration of emerging zoonotic pathogens, while scientific studies and surveillance efforts that focus on this issue remain scarce (17). Knowledge of zoonotic cestodes is limited to the genera *Dibothriocephalus* (diphyllobothriosis), *Hymenolepis,* and *Taenia*, leaving uncommon neglected cestode infections such as bertielliosis, dipylidiasis, echinococcosis, inermicapsiferosis, raillietinosis, mesocestoidiosis, and sparganosis, which are rarely reported clinically and underestimated even by specialists (4, 18, 19). Wild canids comprise a large group of carnivores that are distributed throughout the world, often living in close proximity to human populations (20–22). The Neotropics are home to a total of 10 wild canid species with varied behaviors, habitats, and forms (Table 1). The current study presents the findings on the gastrointestinal helminth parasite in two highly divergent free-ranging Neotropical wild canid (NWC) species: the elusive semiaquatic diurnal/crepuscular bush dog and the nocturnal ground-dwelling crab-eating fox. Furthermore, we examine the potential role that NWC may play as definitive hosts (DH) in the transmission and maintenance of neglected zoonotic helminthiases, providing new insights into this unresolved issue.

**Table 1:** Extant wild canid species distributed in the neotropics.

Genus	Species	Common name	Classification risk ^§^
*Atelocynus*	*microtis*	Short-eared dog	NT
*Cerdocyon*	*thous* ^‡^	Crab-eating fox	LC
*Chrysocyon*	*brachyurus*	Maned wolf	NT
*Speothos*	*venaticus* ^‡^	Bush dog	NT
*Lycalopex*	*vetulus*	Hoary fox	NT
*Lycalopex*	*sechura*	Sechuran fox	NT
*Lycalopex*	*gymnocercus*	Pampa’s fox	LC
*Lycalopex*	*fulvipes*	Darwin’s fox	EN
*Lycalopex*	*culpaeus*	Culpeo	LC
*Lycalopex*	*griseus*	Chilla	LC

^‡^ Wild canid species included in this study. ^§^ Based on the International Union for Conservation of Nature (IUCN) Red List of Threatened Species. EN, endangered. NT, near threatened. LC, least concern.

## 2. Materials and methods

### 2.1. Study areas and sample collection

**Figure 1:**
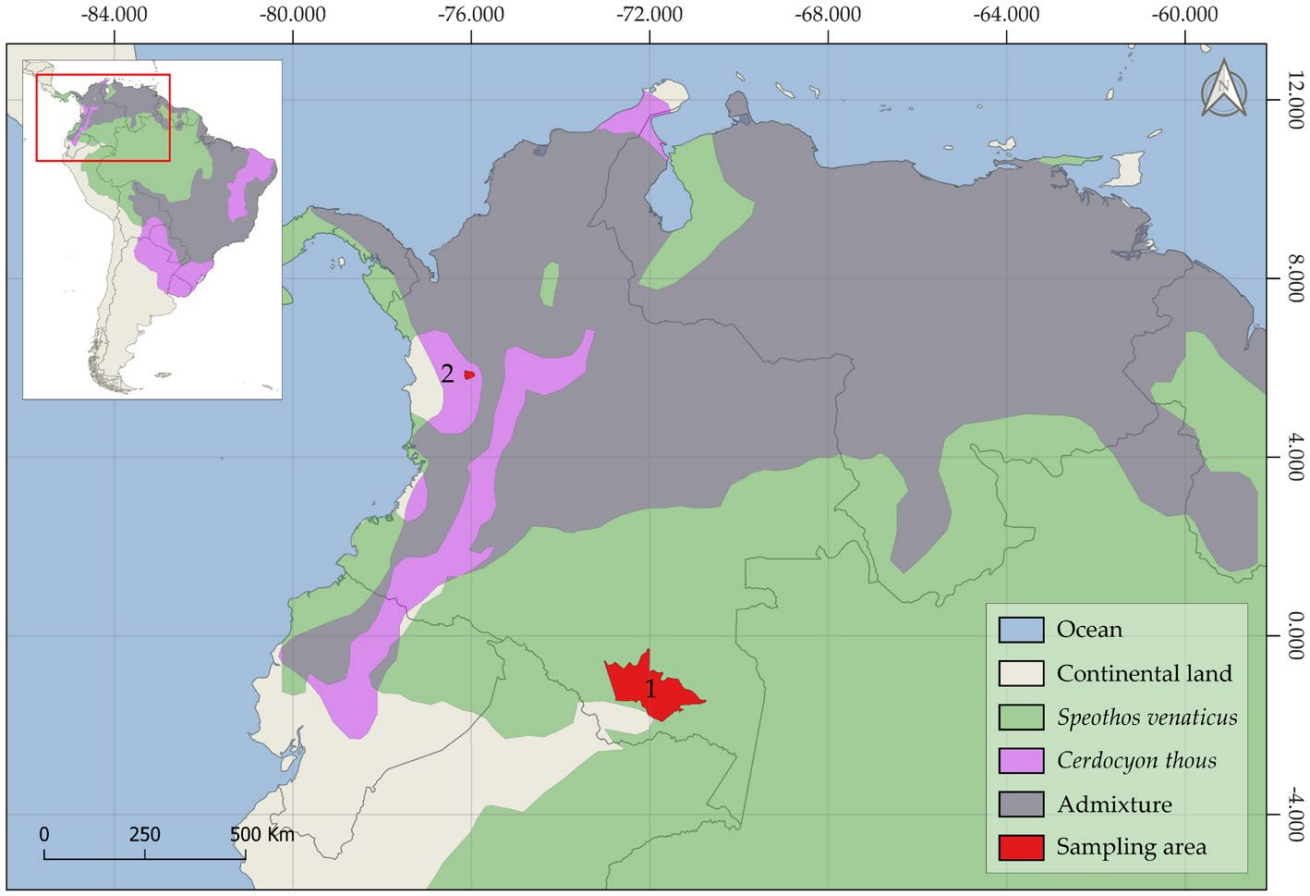
The geographic map depicts the historical distribution range of the bush dog (*Speothos venaticus*), the crab-eating fox (*Cerdocyon thous*), and the admixture zone where both species are found. The Amazonian (1) and Andean (2) sampling areas of this study are shown in red.

Based on the Köppen–Geiger classification system, the Amazonian and Andean sampling areas were found in tropical rainforests and temperate, warm summers without dry climates, respectively (23). Animal sampling was focused on the northern regions of South America (Figure 1) and within the distribution range of bush dogs and crab-eating foxes in the Neotropical area. The current study included samples collected between 2019 and 2021 as part of a national wildlife conservation and monitoring program carried out by veterinarians/mammologists in Colombia. Additionally, animals found dead and collected by indigenous peoples and local communities were included in the study. Due to the evasive behavior of bush dogs, parasite specimens were collected from direct sampling sites on trails that were systematically monitored by trap cameras. The general features and morphometric characteristics of wild carnivore deposits were followed for fecal identification (24). Moreover, associated tracks and local traditional ecological knowledge were also used to sample these elusive individuals, as previously described (25, 26). Therefore, fecal samples were collected as fresh as possible. No bush dog carcasses were examined during the study period. However, scattered proglottids and cestode strobila segments were found partially dehydrated during the macroscopical examination of feces from two monitored bush dogs (*n* = 2) in the Amazonian municipality of Puerto Santander, Colombia. Parasite collection from crab-eating foxes was carried out during the necropsy of a dead animal (*n* = 1) in the Andean municipality of Ciudad Bolívar., Colombia, and in an environment where grass-fed cattle are raised. The head, thoracic, and abdominal cavities were extensively examined for the presence of ecto- and endoparasites. The entire gastrointestinal tract, heart, spleen, kidneys, and respiratory tract were removed and thoroughly inspected *in situ* for the presence of macroscopic parasites using a 40X-25 mm glass magnifier. An adult cestode specimen was carefully recovered from fresh feces collected from the gut lumen after a longitudinal intestinal incision. Fecal samples were then collected directly from the gastrointestinal tract and dry-preserved until examination.

After macroscopic observation of cestode strobila and free proglottids in the feces, all collected parasite specimens were handled using fine entomological tweezers, gently rinsed, washed three times with 0.9% pre-warmed phosphate-buffered saline (PBS), and subsequently preserved in ~96% EtOH until microscopic and molecular evaluation. Combined sedimentation-flotation and modified sodium acetate–acetic acid-formalin standardized parasitological techniques were used to analyze wild canid fecal samples (27). In addition, gravid proglottids obtained from the crab-eating fox tapeworm strobila were dissected and wet mounted on slides. This non-invasive method for fecal collection allowed for the recovery of adult cestode specimens without unnecessary manipulation, trapping, or disturbance of these free-ranging canids (25, 28).

### 2.2. Phenotypic evaluation of adult cestode specimens

General morphologic and morphometric taxonomic traits were observed, and parasite stage identification was conducted under microscopic analysis using an Olympus BX53™ semi-motorized light microscope (Olympus Corporation, Tokyo, Japan) at 400 and 1,000X magnification. The Olympus DP74™ digital camera was used to capture photomicrographs of eggs, adult strobila, and proglottids. Parasites were measured using the cellSens™ standard imaging software. Additionally, cestode proglottids were dehydrated in ethanol series (75, 80, 85, 90, 96, and 100%), transferred to a fixative solution (i.e., formalin, 95% EtOH, glacial acetic acid, glycerine, and Milli-Q ultrapure distilled water; 10:25:5:10:50 parts, respectively), clarified with lactophenol, and stained with Semichon’s acetocarmine. Finally, the proglottids were wet-mounted and Berlese’s fluid-mounted on slides as described previously (29).

### 2.3. Molecular phylogenetics

The complete coding sequence of the cytochrome c oxidase subunit I (*cox1*) gene was amplified in two overlapping fragments with the primers *cox1*F and JB4.5, and JB3 and *cox1*R, respectively (30, 31) using Phusion High-Fidelity DNA Polymerase (New England Biolabs, Inc., Ipswich, USA) and the following cycling conditions: 35 cycles of 10 s at 98°C, 15 s at 50°C (*cox1*F + JB4.5) or 60°C (JB3 + *cox1*R), and 50 s at 72°C. PCR products were gel-checked, purified with Exonuclease I and FastAP alkaline phosphatase (Thermo Fisher Scientific, Waltham, USA), and directly Sanger-sequenced at SeqMe (Dobříš, Czech Republic). Contiguous gene sequences were assembled, visually checked, and trimmed to the *cox1* coding region in Geneious Prime 2020.0.5^1^ and deposited in GenBank under accession numbers OR251823 and OR251823. The resulting sequences were aligned with previously published *cox1* data from *Dipylidium* specimens in addition to other closely related species using MAFFT’s (32) L-INS-i translational align plugin of Geneious. The use of *Nippotaenia chaenogobii* (JQ2685509) and *Nippotaenia mogurndae* (ON640728) as outgroup taxa and the selection of relevant ingroup representatives were based on previous phylogenetic estimates, most notably by Waeschenbach et al. (33) and Guo et al. (34). The phylogenetic tree was estimated under the maximum likelihood criterion in IQ-TREE (35). The best-fitting model of nucleotide evolution was selected according to the corrected Akaike information criterion in IQ-TREE (36), and nodal supports were estimated by running 1,000 standard nonparametric bootstrap replicates and 10,000 repetitions of the SH-like approximated likelihood ratio test.

## 3. Results

### 3.1. Morphological and morphometric parasite identification

**Figure 2:**
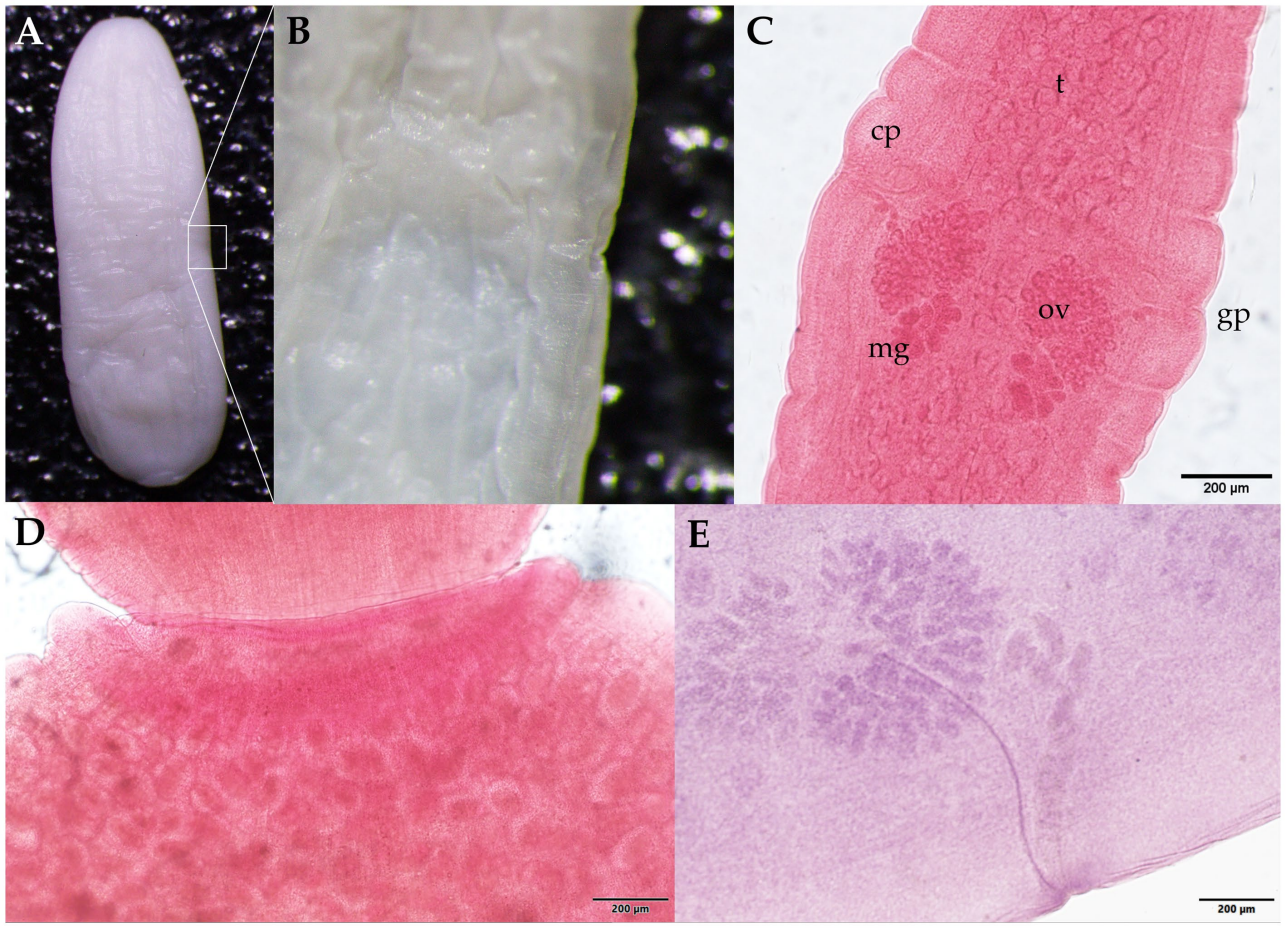
Proglottids of *Dipylidium caninum* s.l. (family Dipylidiidae) collected from the feces of the Amazonian bush dog (*Speothos venaticus*). **(A)** Wet mount unstained gravid proglottid. The white square indicates **(B)** a lateral magnified view of the genital pore. **(C)** Mature proglottids stained with Semichon’s acetocarmine; two sets of symmetrically distributed genital organs are visible, with the testis parenchyma (t), cirrus pouch (cp), genital pore (gp), ovaries (ov), and the Mehlis glands (mg). **(D)** Close-up of the seed-shaped ovigerous proglottid end with round to oval egg capsules (packets) with an average length of 31–50 μm and a width of 27–48 μm (*n* = 88). **(E)** Photomicrograph of the parasite showing details of one of two sets of male and female reproductive organs. Scale bars: **(C–E)** 200 μm.

**Figure 3:**
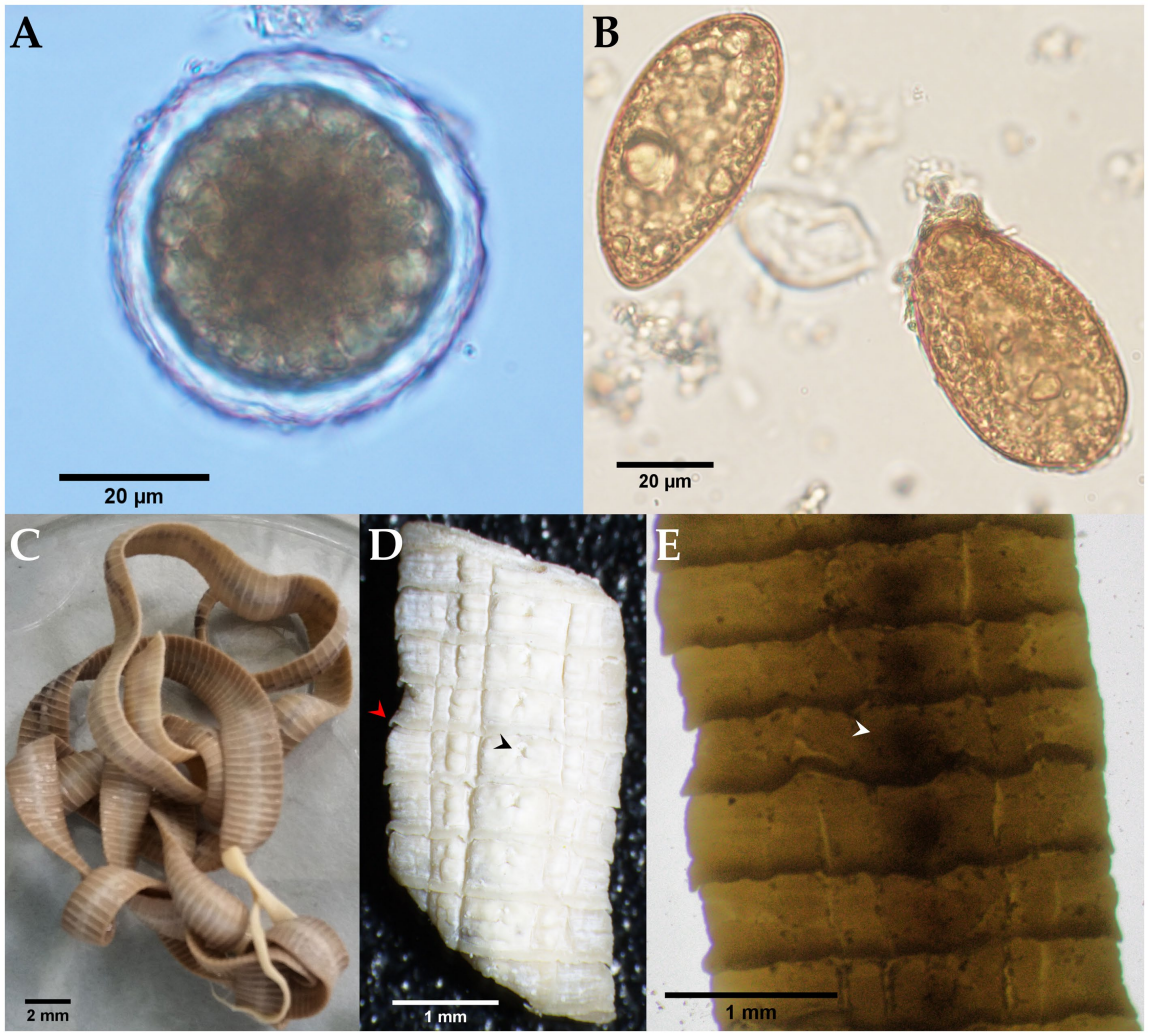
Microscopic and stereomicroscopic morphological examination of parasite stages found in the feces of a free-ranging crab-eating fox (*Cerdocyon thous*). **(A)** Non-embryonated egg of *Lagochilascaris cf. minor* (51.21 × 51.57 μm) with an evident 5.79 μm thick eggshell. **(B)** Yellowish-brown, cone-shaped operculated eggs of *Spirometra mansoni* (61.67 × 34.97 μm). **(C)** Adult *S. mansoni* (from the Andes) with a spoon-shaped scolex and characteristic pink color due to the presence of host vitamin B12. **(D)** Close-up photograph of serrated gravid proglottids (red arrowhead); a genital pore is indicated by the black arrowhead. **(E)** Whole-mounted strobilus segment showing the centrally located spiralled uterus (white arrowhead).

Morphological identification of the whitish, flat, barrel-shaped segments recovered from bush dog feces (Figure 2) was based on observation of the typical longer-than-wide shape, with each proglottid having two bilateral genital pores, one at the center of each lateral margin. The mean gravid proglottid measurements (*n* = 10) were 12.082 mm (SD ± 0.542 mm) in length and 3.996 mm (SD ± 0.344 mm) in width. The phenotypic evaluation corresponds well with *Dipylidium caninum* s.l. (Dipylidiidae). Thin-shelled capsules (ovigerous capsules) containing eggs were also noticed inside gravid proglottids (Figure 2D). Regarding the parasitological evaluation of the crab-eating fox, non-embryonated ascarid-type eggs (51.21 × 51.57 μm) with a thick eggshell and a coarsely pitted surface containing multiple excavations were observed (Figure 3A). The morphological traits of the egg correspond well to *Lagochilascaris minor,* previously described in South American wild carnivore definitive hosts (37, 38). Additionally, parasite stages (i.e., adults and eggs) of the diphyllobothriidean species *Spirometra mansoni* were detected. A weakly muscled, medium-sized, pink-colored cestode (89.73 cm in length) with a long, prominent neck was also recorded. External segmentation of the strobila was noted throughout the specimen. The cestode showed a well-developed spoon-shaped scolex without inrolling bothrial edges. The mature and gravid proglottids were serrated, and the eggs presented a clearly visible unique operculum and an oval shape with a pointed end (Figures 3B–E and Supplementary Video S1). The average proglottid measurements (*n* = 483) were 454.14 μm (SD ± 207.16 μm) in length and 1.78 mm (SD ± 0.73 mm) in width. The strobila segments of this parasite specimen were previously used for the molecular identification of *S. mansoni* reported by Brabec et al. (39).

### 3.2. Molecular characterization of *Dipylidium caninum*

**Figure 4:**
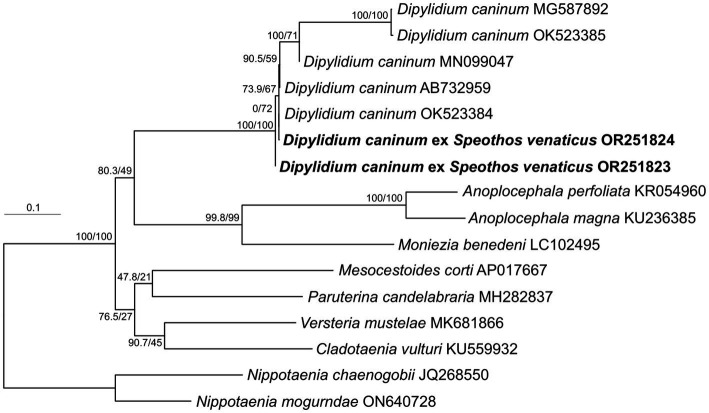
Phylogenetic position of *Dipylidium caninum* isolates obtained from two Amazonian bush dog hosts. Maximum likelihood tree from IQ-TREE based on nearly complete (1,563 bp) *cox1* gene sequences analyzed as a single partition using the TIM + F + R5 model. Nodal values show SH-like approximated likelihood ratio test values (10,000 replicates) and standard nonparametric bootstrap supports (1,000 repetitions). Newly characterized specimens are shown in bold. GenBank accessions are shown after taxon names. The branch length scale bar indicates the number of substitutions per site.

Strobila segments isolated from two separately collected Andean bush dog fecal samples were molecularly characterized by *cox1* sequencing. Maximum likelihood phylogenetic analysis confirmed the species identification as *D*. *caninum*, placing both specimens at the base of a well-defined group composed exclusively of *D*. *caninum* representatives (Figure 4). The lineage of the *D*. *caninum* group consists of two genetically differentiated subgroups corresponding to the previously described canine- and feline-specific genotypes of *D*. *caninum* (40). Within these, five canine-specific genotype representatives formed a relatively basal, non-monophyletic, statistically unsupported assemblage of specimens, while the feline-specific genotype represented by two specimens (MG587892 and OK523385) formed a relatively derived, well-supported internal lineage. The Andean bush dog isolates are grouped basally within the canine-specific genotype representatives (Figure 4).

## 4. Discussion

In an increasingly globalized world, anthropogenic factors have intensified the human-wildlife interface, thus increasing the risk of disease spillover, reduction of biodiversity, and food web collapse (41–43). Among carnivores, only 54.3% of the global species’ distribution range comprises high-quality habitats due to landscape fragmentation and loss of connectivity (44). Nowadays, surveillance of wildlife-derived infectious diseases is imperative to better understand the impact of disease on populations, eco-epidemiology, and biodiversity conservation (45–47). As already stated, synanthropic wild canids have also been reported as natural reservoirs of novel helminth parasites (48–50).

Parasitological surveys of bush dogs are still limited due to their elusive nature and crepuscular behavior, and thus they remain one of the lesser-known wild canid species. Nonetheless, the causative agent of chronic polycystic human echinococcosis (i.e., *Echinococcus vogeli*) was described for the first time in 1972 in a wild bush dog captured in South America (51, 52). Furthermore, other zoonotic-relevant parasites such as *Toxocara canis*, *Lagochilascaris* sp., and *Spirometra* sp. have been reported in wild bush dogs along with the occurrences of *Spirocerca lupi*, *Ancylostoma caninum*, *Taenia* sp., and the apicomplexan *Cystoisospora caninum* (53, 54). Additionally, parasites such as *Dioctophyme renale*, *Dirofilaria immitis*, *Neospora caninum*, *Rangelia vitalii*, the cyst-forming coccidia *Hammondia heydorni,* and the zoonotic parasites *Angiostrongylus cantonensis*, *Dipylidium caninum*, *Leishmania infantum* (syn. *L*. *chagasi*), *Toxoplasma gondii*, and various important ticks have been reported for the crab-eating fox (55–63). Thus, both NWC species may contribute to the environmental maintenance and transmission of human and domestic animal parasitoses. Given the lack of information regarding the occurrence and distribution of helminthiases of public health concern, such as dipylidiasis, lagochilascariosis, and sparganosis, the results presented here collectively provide new insights into the potential of NWC in the emergence and transmission of zoonotic infectious diseases. Here, we successfully perform the identification of neglected zoonotic helminths harbored by bush dogs and crab-eating foxes.

The worldwide occurrence of *D*. *caninum* s.l. in domestic dogs and cats is well documented, as is human dipylidiasis, particularly in young children (64–68). Humans become infected by accidental ingestion of *D*. *caninum*-cysticercoid-carrying intermediate hosts (IH), most commonly fleas or chewing lice (69, 70). Based on molecular techniques that allow for the differentiation of cryptic species and hidden genetic lineages, two genetically distinct lineages, i.e., the so-called *D. caninum* canine and *D*. *caninum* feline genotypes, have been proposed within the genus (40, 71). In contrast to human dipylidiasis, *D*. *caninum* infections rarely produce clinical manifestations in canids or felids. Nonetheless, animals that frequently carry *D*. *caninum*-infected IH contribute to human parasite transmission (72, 73). Wild carnivores such as dingoes (*Canis dingo*), golden jackals (*Canis aureus*), jaguars (*Panthera onca*), red foxes (*Vulpes vulpes*), and spotted hyenas (*Crocuta crocuta*) are wild natural reservoir hosts and thus essential for the maintenance of the parasite life cycle (72, 74–77). *Dipylidium caninum* infections in crab-eating fox populations have been suggested as a possible consequence of anthropogenic expansion into the natural habitats of wild hosts (60). A study in the rural high-mountain region of Colombia reported the occurrence of *D*. *caninum* with an estimated prevalence of 20% (SD ± 8.7%) in free-roaming and peri-domestic dog populations (78). In Colombia, the parasite has only previously been reported in humans and domestic hosts (79). Therefore, to the best of our knowledge, the results presented here expand the geographic distribution range of wildlife dipylidiasis to the Pan-Amazonian and northern Andean regions, providing the first host record for bush dogs. Additionally, we establish here that the analyzed cestode proglottids from bush dogs correspond to the *D*. *caninum* canine genotype, which occurs at a higher frequency in canids, has a shorter pre-patency, and has a longer life span than the *D*. *caninum* feline genotype (40).

The detection of the ascarid nematode genus *Lagochilascaris* raises public health concerns since human lagochilascariosis, mainly due to *L. minor,* is still an extremely neglected zoonotic disease of the Neotropics. The definitive hosts are carnivores (i.e., canids and felids) carrying intestinal adults that shed highly resistant ascarid-like eggs with a thick and rough eggshell (37, 80). Humans acquire lagochilascariosis through the ingestion of infected rodent IH (e.g., agoutis, mice, rats) containing third-stage larvae (L3), but there is also evidence that humans might become infected after ingestion of embryonated eggs of *Lagochilascaris* (81). So far, more than 100 human cases of lagochilascariosis have been recorded in the Americas (80). Three cases of human lagochilascariosis have been documented in the Caribbean/Pacific, and Amazonian regions of Colombia (82, 83). Regardless, the present study constitutes the first non-human report of this parasite in Colombia. Because the amount of DNA obtained from the isolated *Lagochilascaris cf. minor* eggs was extremely low and showed partial degradation, subsequent phylogenetic analysis was not feasible. Nevertheless, surveillance for human lagochilascariosis by local public health authorities should be recommended.

Globally distributed sparganosis is a neglected food- and waterborne zoonotic disease caused by infection with cestodes of the genus *Spirometra* (Diphyllobothriidea), which is frequently reported in numerous wildlife species (14, 18, 39, 84). Sparganosis manifests as muscular and subcutaneous larvae (spargana), but brain invasion has also been reported (85). The obligate heteroxenous parasite life cycle involves carnivores, where intestinal adults shed eggs that are subsequently released into the environment with feces. In the aqueous environment, the eggs hatch into coracidia, which are ingested by copepods as the first IH in which a procercoid larva develops. These larvae are infective to the second tetrapod IH (e.g., frogs, snakes, and birds), where maturation into plerocercoid larvae takes place (86). Humans become infected by eating raw IH flesh, using it in traditional poultices, or drinking water containing infected copepods (87). In South America, a total of 16 human cases of sparganosis have been reported, one of them in Colombia (87, 88). The cestode specimen of the crab-eating fox morphologically described here was molecularly identified as *Spirometra mansoni*, the first report of the species in South America (39). As sparganosis remains one of the least studied diseases, the morphological data presented here support the findings of Brabec et al. in 2022 (39), which effectively enlarged the distribution range of *S. mansoni* for the Neotropics, and call for further investigation of human sparganosis.

Here, we have provided evidence for important zoonotic helminth infections in highly divergent free-ranging NWC species. Consequently, investigation of the potential role of the elusive semiaquatic bush dog, the synanthropic/peri-domestic crab-eating fox, and other poorly studied NWCs in the transmission cycle of these parasites to humans seems essential. Additionally, future ectoparasite research on different lice and flea taxa (e.g., *Felicola subrostratus*, *Trichodectes canis*, Archaeopsyllinae, and Pulicinae subfamilies) infesting wild carnivore populations is urgently needed to identify the IH harboring *D. caninum* cysticercoids and thus enabling zoonotic transmission of dipylidiasis. The consequences of coinfections on epidemiology and host fitness require better knowledge of NWC-associated infectious agents to understand their role in the emergence of dipylidiasis, lagochilascariosis, and sparganosis (89). Based on these findings, we encourage further parasitologic investigations to be conducted among NWCs, specifically regarding their endo- and ectoparasites. In conclusion, an ongoing parasitological survey of wildlife is critical for implementing public health strategies to avoid zoonotic spillover in a pathogen-related surveillance network.

## Data availability statement

The datasets presented in this study can be found in online repositories. The names of the repository/repositories and accession number(s) can be found at: https://www.ncbi.nlm.nih.gov/nuccore/OR251824, OR251824; and https://www.ncbi.nlm.nih.gov/nuccore/OR251823, OR251823.

## Ethics statement

The animal study was approved by the Ethics Committee for Animal Experimentation (CEEA) of the Universidad de Antioquia, Colombia (AS No. 132) under collection permit No. 0524 of 2014 (IDB0321), procedures were conducted according to the Guidelines of the American Society of Mammologists for the use of wild mammals in research and education, and the EU Directive 2010/63/EU. The study was conducted in accordance with the local legislation and institutional requirements.

## Author contributions

MU: conceptualization, investigation, writing-original draft preparation. MU and JB: methodology, software, and visualization. JB, JC-G, and CH: validation. JB and MU: formal analysis. CH and JC-G: resources and funding acquisition. JB, CH, and JC-G: data curation and writing – review and editing. JC-G and CH: supervision. All authors have read and agreed to the published version of the manuscript.

## Funding

The project in which the samples were collected was funded by the CIBAV Research Group-Centro de Investigaciones Básicas y Aplicadas en Veterinaria (COL0153246), Universidad de Antioquia, Consolidation Strategy 2018–2019. The molecular characterization of the specimens was funded by the Czech Science Foundation, Project No. 19-28399X. The APC was funded by Justus Liebig University in Giessen. We would like to extend our thanks to the Bicentennial Doctoral Excellence Scholarship Program of Colombia (“Programa de becas de excelencia doctoral del bicentenario”) for the financial support to the Ph.D. student MU.

## Conflict of interest

The authors declare that the research was conducted in the absence of any commercial or financial relationships that could be construed as a potential conflict of interest.

## Publisher’s note

All claims expressed in this article are solely those of the authors and do not necessarily represent those of their affiliated organizations, or those of the publisher, the editors and the reviewers. Any product that may be evaluated in this article, or claim that may be made by its manufacturer, is not guaranteed or endorsed by the publisher.

## Footnotes

^1^ http://www.geneious.com

## References

[1] Rahman MT Sobur MA Islam MS Ievy S Hossain MJ El Zowalaty ME. Zoonotic diseases: etiology, impact, and control. Microorganisms. (2020) 8:1405. doi: 10.3390/microorganisms8091405, PMID: 32932606 PMC7563794

[2] Salyer SJ Silver R Simone K Barton BC. Prioritizing zoonoses for global health capacity building—themes from one health zoonotic disease workshops in 7 countries, 2014–2016. Emerg Infect Dis. (2017) 23:S55–64. doi: 10.3201/eid2313.170418, PMID: 29155664 PMC5711306

[3] Xiao N Yao J-W Ding W Giraudoux P Craig PS Ito A. Priorities for research and control of cestode zoonoses in Asia. Infect Dis Poverty. (2013) 2:16. doi: 10.1186/2049-9957-2-16, PMID: 23915395 PMC3750256

[4] Sapp SGH Bradbury RS. The forgotten exotic tapeworms: a review of uncommon zoonotic Cyclophyllidea. Parasitology. (2020) 147:533–58. doi: 10.1017/S003118202000013X, PMID: 32048575 PMC7174715

[5] Irie T Uraguchi K Ito T Yamazaki A Takai S Yagi K. First report of *Sarcocystis pilosa* sporocysts in feces from red fox, *Vulpes vulpes schrencki*, in Hokkaido, Japan. Int J Parasitol Parasites Wildl. (2020) 11:29–31. doi: 10.1016/j.ijppaw.2019.12.001, PMID: 31879592 PMC6920301

[6] Elmore SA Lalonde LF Samelius G Alisauskas RT Gajadhar AA Jenkins EJ. Endoparasites in the feces of arctic foxes in a terrestrial ecosystem in Canada. Int J Parasitol Parasites Wildl. (2013) 2:90–6. doi: 10.1016/j.ijppaw.2013.02.005 24533320 PMC3862500

[7] Otranto D Deplazes P. Zoonotic nematodes of wild carnivores. Int J Parasitol Parasites Wildl. (2019) 9:370–83. doi: 10.1016/j.ijppaw.2018.12.011, PMID: 31338295 PMC6626844

[8] Myšková E Brož M Fuglei E Kvičerová J Mácová A Sak B. Gastrointestinal parasites of arctic foxes (*Vulpes lagopus*) and sibling voles (*Microtus levis*) in Spitsbergen. Svalbard *Parasitol Res*. (2019) 118:3409–18. doi: 10.1007/s00436-019-06502-8, PMID: 31729572

[9] Duscher GG Leschnik M Fuehrer H-P Joachim A. Wildlife reservoirs for vector-borne canine, feline and zoonotic infections in Austria. Int J Parasitol Parasites Wildl. (2015) 4:88–96. doi: 10.1016/j.ijppaw.2014.12.001, PMID: 25830102 PMC4356739

[10] Karamon J Samorek-Pieróg M Sroka J Bilska-Zając E Dąbrowska J Kochanowski M. The first record of *Echinococcus ortleppi* (G5) tapeworms in grey wolf (*Canis lupus*). Pathogens. (2021) 10:853. doi: 10.3390/pathogens10070853, PMID: 34358003 PMC8308913

[11] Macchioni F Coppola F Furzi F Gabrielli S Baldanti S Boni CB. Taeniid cestodes in a wolf pack living in a highly anthropic hilly agro-ecosystem. Parasite. (2021) 28:10. doi: 10.1051/parasite/2021008, PMID: 33544075 PMC7863970

[12] Oudni-M’rad M Chaâbane-Banaoues R M’rad S Trifa F Mezhoud H Babba H. Gastrointestinal parasites of canids, a latent risk to human health in Tunisia. Parasit Vectors. (2017) 10:280. doi: 10.1186/s13071-017-2208-3, PMID: 28583158 PMC5460421

[13] Guerra D Armua-Fernandez MT Silva M Bravo I Santos N Deplazes P. Taeniid species of the Iberian wolf (*Canis lupus signatus*) in Portugal with special focus on *Echinococcus* spp. Int J Parasitol Parasites Wildl. (2013) 2:50–3. doi: 10.1016/j.ijppaw.2012.11.007, PMID: 24533315 PMC3862541

[14] Bagrade G Králová-Hromadová I Bazsalovicsová E Radačovská A Kołodziej-Sobocińska M. The first records of *Spirometra erinaceieuropaei* (Cestoda: Diphyllobothriidae), a causative agent of human sparganosis, in Latvian wildlife. Parasitol Res. (2021) 120:365–71. doi: 10.1007/s00436-020-06957-0 33174072 PMC7846523

[15] Allen T Murray KA Zambrana-Torrelio C Morse SS Rondinini C Di Marco M. Global hotspots and correlates of emerging zoonotic diseases. Nat Commun. (2017) 8:1124. doi: 10.1038/s41467-017-00923-8, PMID: 29066781 PMC5654761

[16] Namusisi S Mahero M Travis D Pelican K Robertson C Mugisha L. A descriptive study of zoonotic disease risk at the human-wildlife interface in a biodiversity hot spot in South Western Uganda. PLoS Negl Trop Dis. (2021) 15:e0008633. doi: 10.1371/journal.pntd.0008633, PMID: 33406074 PMC7845987

[17] Jones KE Patel NG Levy MA Storeygard A Balk D Gittleman JL. Global trends in emerging infectious diseases. Nature. (2008) 451:990–3. doi: 10.1038/nature06536, PMID: 18288193 PMC5960580

[18] Kuchta R Kołodziej-Sobocińska M Brabec J Młocicki D Sałamatin R Scholz T. Sparganosis (*Spirometra*) in Europe in the molecular era. Clin Infect Dis. (2021) 72:882–90. doi: 10.1093/cid/ciaa1036, PMID: 32702118

[19] Dhaliwal BBS Juyal PD. Cestode zoonoses In: . Parasitic Zoonose*s*. New Delhi: Springer India (2013). 65–82. doi: 10.1007/978-81-322-1551-6

[20] Schipper J Chanson JS Chiozza F Cox NA Hoffmann M Katariya V. The status of the world’s land and marine mammals: diversity, threat, and knowledge. Science. (2008) 322:225–30. doi: 10.1126/science.1165115 18845749

[21] Brandão EMV Xavier SCC Rocha FL Lima CFM Candeias ÍZ Lemos FG. Wild and domestic canids and their interactions in the transmission cycles of Trypanosoma Cruzi and Leishmania spp. in an area of the Brazilian Cerrado. Pathogens. (2020) 9:818. doi: 10.3390/pathogens9100818, PMID: 33036238 PMC7600672

[22] Macpherson CNL. Human behaviour and the epidemiology of parasitic zoonoses. Int J Parasitol. (2005) 35:1319–31. doi: 10.1016/j.ijpara.2005.06.004 16102769

[23] Beck HE Zimmermann NE McVicar TR Vergopolan N Berg A Wood EF. Present and future Köppen-Geiger climate classification maps at 1-km resolution. Sci Data. (2018) 5:180214. doi: 10.1038/sdata.2018.214, PMID: 30375988 PMC6207062

[24] Chame M. Terrestrial mammal feces: a morphometric summary and description. Mem Inst Oswaldo Cruz. (2003) 98:71–94. doi: 10.1590/S0074-02762003000900014, PMID: 12687767

[25] Uribe M Payán E Brabec J Vélez J Taubert A Chaparro-Gutiérrez JJ. Intestinal parasites of neotropical wild jaguars, pumas, ocelots, and jaguarundis in Colombia: old friends brought back from oblivion and new insights. Pathogens. (2021) 10:822. doi: 10.3390/pathogens10070822, PMID: 34209062 PMC8308835

[26] Zuercher GL Gipson PS Stewart GC. Identification of carnivore feces by local peoples and molecular analyses. Wildl Soc Bull. (2003) 31:961–70.

[27] Yang J Scholten T. A fixative for intestinal parasites permitting the use of concentration and permanent staining procedures. Am J Clin Pathol. (1977) 67:300–4. doi: 10.1093/ajcp/67.3.300, PMID: 65913

[28] Uribe M Hermosilla C Rodríguez-Durán A Vélez J López-Osorio S Chaparro-Gutiérrez JJ. Parasites circulating in wild synanthropic capybaras (*Hydrochoerus hydrochaeris*): a one health approach. Pathogens. (2021) 10:1152. doi: 10.3390/pathogens10091152, PMID: 34578184 PMC8467752

[29] Swan DC. Berlese’s fluid: remarks upon its preparation and use as a mounting medium. Bull Entomol Res. (1936) 27:389–91. doi: 10.1017/S0007485300058259

[30] Bowles J Blair D McManus DP. Genetic variants within the genus *Echinococcus* identified by mitochondrial DNA sequencing. Mol Biochem Parasitol. (1992) 54:165–73. doi: 10.1016/0166-6851(92)90109-W, PMID: 1435857

[31] Wicht B Yanagida T Scholz T Ito A Jiménez JA Brabec J. Multiplex PCR for differential identification of broad tapeworms (Cestoda: Diphyllobothrium) infecting humans. J Clin Microbiol. (2010) 48:3111–6. doi: 10.1128/JCM.00445-10, PMID: 20592146 PMC2937707

[32] Katoh K Standley DM. MAFFT multiple sequence alignment software version 7: improvements in performance and usability. Mol Biol Evol. (2013) 30:772–80. doi: 10.1093/molbev/mst010, PMID: 23329690 PMC3603318

[33] Waeschenbach A Webster BL Littlewood DTJ. Adding resolution to ordinal level relationships of tapeworms (Platyhelminthes: Cestoda) with large fragments of mtDNA. Mol Phylogenet Evol. (2012) 63:834–47. doi: 10.1016/j.ympev.2012.02.020, PMID: 22406529

[34] Guo X Liu J Hao G Zhang L Mao K Wang X. Plastome phylogeny and early diversification of Brassicaceae. BMC Genomics. (2017) 18:176. doi: 10.1186/s12864-017-3555-3, PMID: 28209119 PMC5312533

[35] Nguyen L-T Schmidt HA von Haeseler A Minh BQ. IQ-TREE: a fast and effective stochastic algorithm for estimating maximum-likelihood phylogenies. Mol Biol Evol. (2015) 32:268–74. doi: 10.1093/molbev/msu300 25371430 PMC4271533

[36] Kalyaanamoorthy S Minh BQ Wong TKF von Haeseler A Jermiin LS. ModelFinder: fast model selection for accurate phylogenetic estimates. Nat Methods. (2017) 14:587–9. doi: 10.1038/nmeth.4285, PMID: 28481363 PMC5453245

[37] Rodriguez-Vivas RI Salazar-Grosskelwing E Ojeda-Chi MM Flota-Burgos GJ Solano-Barquero A Trinidad-Martínez I. First morphological and molecular report of Lagochilascaris minor (Nematoda, Ascarididae) in a domestic cat from Veracruz, Mexico. Vet Parasitol (Amst). (2023) 37:100823. doi: 10.1016/j.vprsr.2022.100823, PMID: 36623903

[38] Trindade MAC MRP DM Drehmer CJ Muller G. First record of *Lagochilascaris minor* (Nematoda: Ascarididae) in *Leopardus geoffroyi* (Carnivora: Felidae) in Brazil. Rev Bras Parasitol Veterinária. (2019) 28:812–5. doi: 10.1590/s1984-29612019087, PMID: 31721930

[39] Brabec J Uribe M Chaparro-Gutiérrez JJ Hermosilla C. Presence of *Spirometra mansoni*, causative agent of sparganosis, in South America. Emerg Infect Dis. (2022) 28:2347–50. doi: 10.3201/eid2811.220529, PMID: 36286232 PMC9622250

[40] Beugnet F Labuschagne M Vos C Crafford D Fourie J. Analysis of *Dipylidium caninum* tapeworms from dogs and cats, or their respective fleas. Parasite. (2018) 25:31. doi: 10.1051/parasite/2018029, PMID: 29806593 PMC6013090

[41] Fricke EC Hsieh C Middleton O Gorczynski D Cappello CD Sanisidro O. Collapse of terrestrial mammal food webs since the late Pleistocene. Science. (2022) 377:1008–11. doi: 10.1126/science.abn4012 36007038

[42] Magouras I Brookes VJ Jori F Martin A Pfeiffer DU Dürr S. Emerging zoonotic diseases: should we rethink the animal–human interface? Front Vet Sci. (2020) 7:582743. doi: 10.3389/fvets.2020.582743, PMID: 33195602 PMC7642492

[43] El Bizri HR Morcatty TQ Valsecchi J Mayor P Ribeiro JES Vasconcelos Neto CFA. Urban wild meat consumption and trade in Central Amazonia. Conserv Biol. (2020) 34:438–48. doi: 10.1111/cobi.13420, PMID: 31538670

[44] Crooks KR Burdett CL Theobald DM Rondinini C Boitani L. Global patterns of fragmentation and connectivity of mammalian carnivore habitat. Philos Trans R Soc B Biol Sci. (2011) 366:2642–51. doi: 10.1098/rstb.2011.0120, PMID: 21844043 PMC3140740

[45] Grogan LF Berger L Rose K Grillo V Cashins SD Skerratt LF. Surveillance for emerging biodiversity diseases of wildlife. PLoS Pathog. (2014) 10:e1004015. doi: 10.1371/journal.ppat.1004015, PMID: 24875394 PMC4038591

[46] Martinez ME. The calendar of epidemics: seasonal cycles of infectious diseases. PLoS Pathog. (2018) 14:e1007327. doi: 10.1371/journal.ppat.1007327, PMID: 30408114 PMC6224126

[47] Uribe M Rodríguez-Posada ME Ramirez-Nieto GC. Molecular evidence of orthomyxovirus presence in Colombian Neotropical bats. Front Microbiol. (2022) 13:845546. doi: 10.3389/fmicb.2022.845546, PMID: 35558106 PMC9087557

[48] Nascimento Gomes AP dos Santos MM Olifiers N do Val Vilela R Guimarães Beltrão M Maldonado Júnior A. Molecular phylogenetic study in Spirocercidae (Nematoda) with description of a new species Spirobakerus sagittalis sp. nov. in wild canid *Cerdocyon thous* from Brazil. Parasitol Res. (2021) 120:1713–25. doi: 10.1007/s00436-021-07106-x, PMID: 33693988

[49] Rojas A Sanchis-Monsonís G Alić A Hodžić A Otranto D Yasur-Landau D. *Spirocerca vulpis* sp. nov. (Spiruridae: Spirocercidae): description of a new nematode species of the red fox, *Vulpes vulpes* (Carnivora: Canidae). Parasitology. (2018) 145:1917–28. doi: 10.1017/S0031182018000707, PMID: 29781422

[50] Gomes APN Olifiers N Souza JGR Barbosa HS D’Andrea PS Maldonado A. A new acanthocephalan species (Archiacanthocephala: Oligacanthorhynchidae) from the crab-eating fox (*Cerdocyon thous*) in the Brazilian Pantanal wetlands. J Parasitol. (2015) 101:74–9. doi: 10.1645/13-321.1, PMID: 25291295

[51] Rausch RL Bernstein JJ. *Echinococcus vogeli* sp. n. (Cestoda: Taeniidae) from the bush dog, Speothos venaticus (Lund). Z Tropenmed Parasitol. (1972) 23:25–34. 5050528

[52] Tappe D Stich A Frosch M. Emergence of polycystic neotropical echinococcosis. Emerg Infect Dis. (2008) 14:292–7. doi: 10.3201/eid1402.070742 18258123 PMC2600197

[53] Rinas MA Nesnek R Kinsella JM DeMatteo KE. Fatal aortic aneurysm and rupture in a neotropical bush dog (*Speothos venaticus*) caused by *Spirocerca lupi*. Vet Parasitol. (2009) 164:347–9. doi: 10.1016/j.vetpar.2009.05.006, PMID: 19515493

[54] Vizcaychipi KA Rinas M Irazu L Miyagi A Argüelles CF Dematteo KE. Neotropical zoonotic parasites in bush dogs (*Speothos venaticus*) from upper Paraná Atlantic forests in Misiones, Argentina. Vector-Borne Zoonotic Dis. (2016) 16:664–72. doi: 10.1089/vbz.2015.1929, PMID: 27603553

[55] Ribeiro CT Verocal GG Tavares LER. *Dioctophyme renale* (Nematoda, Dioctophymatidae) infection in the crab-eating fox (*Cerdocyon thous*) from Brazil. J Wildl Dis. (2009) 45:248–50. doi: 10.7589/0090-3558-45.1.248, PMID: 19204359

[56] Almeida Kim PCP Melo Nogueira JF Martins FDC Garcia JL. *Neospora caninum* DNA in feces of crab-eating fox (*Cerdocyon thous* – Linnaeus, 1776) from northeastern Brazil. Acta Trop (2019) 197:105068. doi: 10.1016/j.actatropica.2019.105068, PMID: 31226252

[57] Copat B Bastiani PV Castellarin Jaconi F Wallyson Damarem W Streck AF de Oliveira EC. Presentation of hemolytic and hemorrhagic rangeliosis in *Cerdocyon thous*. Ticks Tick Borne Dis. (2019) 10:690–3. doi: 10.1016/j.ttbdis.2019.02.010, PMID: 30852178

[58] Soares RM Cortez LRPB Gennari SM Sercundes MK Keid LB Pena HFJ. Crab-eating fox (*Cerdocyon thous*), a south American canid, as a definitive host for *Hammondia heydorni*. Vet Parasitol. (2009) 162:46–50. doi: 10.1016/j.vetpar.2009.02.003, PMID: 19303215

[59] Caprioli RA De Andrade CP Argenta FF Ehlers LP Soares JF Pavarini SP. Angiostrongylosis in *Cerdocyon thous* (crab-eating fox) and *Lycalopex gymnocercus* (pampas fox) in southern Brazil. Parasitology. (2019) 146:617–24. doi: 10.1017/S0031182018001865, PMID: 30394242

[60] Vieira FM Luque JL de Souza LS de Moraes Neto AHA Muniz-Pereira LC. *Dipylidium caninum* (Cyclophyllidea, Dipylidiidae) in a wild carnivore from Brazil. J Wildl Dis. (2012) 48:233–4. doi: 10.7589/0090-3558-48.1.233, PMID: 22247400

[61] Almeida JC Melo RPB Kim PCP Guerra NR Alves LC Costa DF. Molecular and serological investigation of infectious diseases in captive and free-range crab-eating fox (*Cerdocyon thous* - Linnaeus, 1776) from northeastern Brazil. Acta Parasitol. (2018) 63:184–9. doi: 10.1515/ap-2018-0021, PMID: 29351073

[62] Ramos VN Lemos FG Azevedo FC Arrais RC Lima CFM Candeias IZ. Wild carnivores, domestic dogs and ticks: shared parasitism in the Brazilian Cerrado. Parasitology. (2020) 147:689–98. doi: 10.1017/S0031182020000335, PMID: 32102697 PMC10317637

[63] Fiorello CV Robbins RG Maffei L Wade SE. Parasites of free-ranging small canids and felids in the Bolivian Chaco. J Zoo Wildl Med. (2006) 37:130–4. doi: 10.1638/05-075.1, PMID: 17312790

[64] Ilić T Nišavić U Gajić B Nenadović K Ristić M Stanojević D. Prevalence of intestinal parasites in dogs from public shelters in Serbia. Comp Immunol Microbiol Infect Dis. (2021) 76:101653. doi: 10.1016/j.cimid.2021.101653, PMID: 33930631

[65] Felsmann M Michalski M Felsmann M Sokół R Szarek J Strzyżewska-Worotyńska E. Invasive forms of canine endoparasites as a potential threat to public health – a review and own studies. Ann Agric Environ Med. (2017) 24:245–9. doi: 10.5604/12321966.1235019, PMID: 28664702

[66] Dantas-Torres F Otranto D. Dogs, cats, parasites, and humans in Brazil: opening the black box. Parasit Vectors. (2014) 7:22. doi: 10.1186/1756-3305-7-22, PMID: 24423244 PMC3914713

[67] Mulinge E Zeyhle E Mpario J Mugo M Nungari L Ngugi B. A survey of intestinal helminths in domestic dogs in a human–animal–environmental interface: the Oloisukut conservancy, Narok County, Kenya. J Helminthol. (2021) 95:e59. doi: 10.1017/S0022149X21000547, PMID: 34641982

[68] Yu Z Ruan Y Zhou M Chen S Zhang Y Wang L. Prevalence of intestinal parasites in companion dogs with diarrhea in Beijing, China, and genetic characteristics of *Giardia* and *Cryptosporidium* species. Parasitol Res. (2018) 117:35–43. doi: 10.1007/s00436-017-5631-7, PMID: 29150700 PMC7088013

[69] Pilarczyk BM Tomza-Marciniak AK Pilarczyk R Rząd I Bąkowska MJ Udała JM. Infection of raccoon dogs (*Nyctereutes procyonoides*) from northern Poland with gastrointestinal parasites as a potential threat to human health. J Clin Med. (2022) 11:1277. doi: 10.3390/jcm11051277, PMID: 35268368 PMC8910989

[70] Darabi E Beigom Kia E Mohebali M Mobedi I Zahabiun F Zarei Z. Gastrointestinal helminthic parasites of stray cats (*Felis catus*) in Northwest Iran. Iran J Parasitol. (2021) 16:418–25. doi: 10.18502/ijpa.v16i3.7095, PMID: 34630587 PMC8476725

[71] Labuschagne M Beugnet F Rehbein S Guillot J Fourie J Crafford D. Analysis of *Dipylidium caninum* tapeworms from dogs and cats, or their respective fleas. Parasite. (2018) 25:30. doi: 10.1051/parasite/2018028, PMID: 29806592 PMC6013089

[72] García-Agudo L García-Martos P Rodríguez-Iglesias M. *Dipylidium caninum* infection in an infant: a rare case report and literature review. Asian Pac J Trop Biomed. (2014) 4:S565–7. doi: 10.12980/APJTB.4.2014APJTB-2014-0034

[73] Hogan CA Schwenk H. *Dipylidium caninum* infection. N Engl J Med. (2019) 380:e39. doi: 10.1056/NEJMicm1813985 31116922

[74] Smout F Skerratt L Johnson C Butler J Congdon B. Zoonotic helminth diseases in dogs and dingoes utilising shared resources in an Australian aboriginal community. Trop Med Infect Dis. (2018) 3:110. doi: 10.3390/tropicalmed3040110, PMID: 30297603 PMC6306763

[75] Ćirović D Pavlović I Penezić A Kulišić Z Selaković S. Levels of infection of intestinal helminth species in the golden jackal *Canis aureus* from Serbia. J Helminthol. (2015) 89:28–33. doi: 10.1017/S0022149X13000552, PMID: 23941681

[76] East ML Kurze C Wilhelm K Benhaiem S Hofer H. Factors influencing *Dipylidium* sp. infection in a free-ranging social carnivore, the spotted hyaena (*Crocuta crocuta*). Int J Parasitol Parasites Wildl. (2013) 2:257–65. doi: 10.1016/j.ijppaw.2013.09.003, PMID: 24533344 PMC3862517

[77] Erol U Sarimehmetoglu O Utuk AE. Intestinal system helminths of red foxes and molecular characterization Taeniid cestodes. Parasitol Res. (2021) 120:2847–54. doi: 10.1007/s00436-021-07227-3, PMID: 34232387

[78] Peña-Quistial MG Benavides-Montaño JA Duque NJR Benavides-Montaño GA. Prevalence and associated risk factors of intestinal parasites in rural high-mountain communities of the Valle del Cauca—Colombia. PLoS Negl Trop Dis. (2020) 14:e0008734. doi: 10.1371/journal.pntd.0008734, PMID: 33035233 PMC7591239

[79] Rousseau J Castro A Novo T Maia C. *Dipylidium caninum* in the twenty-first century: epidemiological studies and reported cases in companion animals and humans. Parasit Vectors. (2022) 15:131. doi: 10.1186/s13071-022-05243-5, PMID: 35534908 PMC9088078

[80] Campos DMB Barbosa AP de Oliveira JA Tavares GG Cravo PVL Ostermayer AL. Human lagochilascariasis—a rare helminthic disease. PLoS Negl Trop Dis. (2017) 11:e0005510. doi: 10.1371/journal.pntd.0005510 28640884 PMC5480834

[81] Scioscia NP Olmos L Gorosábel A Bernad L Pedrana J Denegri GM. Natural infection in pampas fox (*Lycalopex gymnocercus*) by Lagochilascaris major Leiper, 1910 (Nematoda: Ascarididae) in Buenos Aires, Argentina. Zeitschrift für Parasitenkunde (Berlin, Germany). (2018) 117:3023–7. doi: 10.1007/s00436-018-5978-4, PMID: 29938376

[82] Moncada LI Alvarez CA Castellanos C Caceres E Nicholls S Corredor A. *Lagochilascaris minor* in a patient from the Colombian amazon: a case report. Rev Inst Med Trop Sao Paulo. (1998) 40:387–9. doi: 10.1590/s0036-46651998000600009, PMID: 10436660

[83] Little MD Botero D. Two cases of human *Lagochilascaris* infection in Colombia. Am J Trop Med Hyg. (1984) 33:381–6. doi: 10.4269/ajtmh.1984.33.381, PMID: 6539572

[84] Scholz T Kuchta R Brabec J. Broad tapeworms (Diphyllobothriidae), parasites of wildlife and humans: recent progress and future challenges. Int J Parasitol Parasites Wildl. (2019) 9:359–69. doi: 10.1016/j.ijppaw.2019.02.001 31341771 PMC6630034

[85] Hwang Y-H Son W Kim Y-W Kang D-H Chang H-H Goo Y-K. A retrieved sparganum of *Spirometra erinaceieuropaei* from a Korean man during mechanical thrombectomy. Korean J Parasitol. (2020) 58:309–13. doi: 10.3347/kjp.2020.58.3.309, PMID: 32615744 PMC7338899

[86] Mueller JF. The biology of *Spirometra*. J Parasitol. (1974) 60:2–14. doi: 10.2307/3278670 4592501

[87] Kuchta R Scholz T Brabec J Narduzzi-Wicht B. Chapter 17: *Diphyllobothrium*, *Diplogonoporus* and *Spirometra* In: Xiao L Ryan U Feng Y, editors. Biology of foodborne parasites. Section III: Important foodborne helminths. Boca Raton: CRC Press (2015). 299–326.

[88] Gomez JJ Botero D. The first case of sparganosis in Colombia. Am J Trop Med Hyg. (1958) 7:597–9. doi: 10.4269/ajtmh.1958.7.597 13595201

[89] Hoarau AOG Mavingui P Lebarbenchon C. Coinfections in wildlife: focus on a neglected aspect of infectious disease epidemiology. PLoS Pathog. (2020) 16:e1008790. doi: 10.1371/journal.ppat.1008790, PMID: 32881983 PMC7470396

